# Design and Fabrication of Sustained Bacterial Release Scaffolds to Support the Microbiome

**DOI:** 10.3390/pharmaceutics16081066

**Published:** 2024-08-14

**Authors:** Anne Marie Klein, Nanang Qosim, Gareth Williams, Mohan Edirisinghe, Rupy Kaur Matharu

**Affiliations:** 1 Department of Mechanical Engineering, University College London, Torrington Place, London WC1E 7JE, UKm.edirisinghe@ucl.ac.uk (M.E.); 2 Department of Mechanical Engineering, Politeknik Negeri Malang, Jl. Soekarno Hatta No. 9, Malang 65141, Jawa Timur, Indonesia; 3 UCL School of Pharmacy, University College London, 29–39 Brunswick Square, London WC1N 1AX, UK; g.williams@ucl.ac.uk; 4 Department of Civil, Environmental & Geomatic Engineering, University College London, Chadwick Building, Gower Street, London WC1E 6BT, UK

**Keywords:** probiotics, drug delivery, microfibres, bioactive fibres, sustained release

## Abstract

Fibres in the micro- and nanometre scale are suited to a broad range of applications, including drug delivery and tissue engineering. Electrospinning is the manufacturing method of choice, but it has some limitations. Novel pressure-driven fibre-forming techniques, like pressurised gyration (PG), overcome these limitations; however, the compatibility of PG with biological materials has not yet been evaluated in detail. For the first time, this limitation of PG was investigated by optimising PG for microbial cell processing and incorporating bacterial cultures into fibrous polymeric scaffolds for sustained release. Multiple polymer–solvent systems were trialled, including polyvinylpyrrolidone (PVP)/phosphate-buffered saline (PBS) 25% *w*/*v*, polyethylene oxide (PEO)/PBS 20% *w*/*v*, and PVP/ethanol 20% *w*/*v*. Rheological studies revealed the surface tension of the PVP/PBS, PEO/PBS, and PVP/ethanol polymer–solvent systems to be 73.2, 73.9, and 22.6 mN/m, respectively. Scanning electron microscopy showed the median fibre diameters to be between 9.8 μm and 26.1 μm, with PVP producing larger fibres. Overnight *Bacillus subtilis* cultures were then incorporated into the chosen polymeric solutions and processed into fibres using PG. The produced cell-loaded fibres were incubated in LB broth to assess the cell viability of the encapsulated cells. Colony counts post-incubation showed the PVP/PBS 25% fibres resulted in 60% bacterial growth, and PEO/PBS 20% fibres led to 47% bacterial growth, whereas PVP/ethanol 20% fibres did not lead to any bacterial growth. Based on the results gathered during this study, it can be concluded that PG offers a promising way of encapsulating cells and other sensitive biological products while having many notable advantages compared to electrospinning. This research demonstrates proof of concept research-based evidence and showcases the potential of pressurised gyration as a key disruptive innovation in probiotic delivery system design and manufacturing.

## 1. Introduction

Fibrous scaffolds have gained much popularity for a broad range of bioengineering applications, including wound healing, tissue engineering, and drug delivery [1]. These scaffolds are usually composed of continuous polymeric fibres with outer diameters in the nano- or micrometre range. Additionally, fibre orientation can either be random or aligned to suit the intended end use. The architecture of fibrous scaffolds gives rise to many advantageous properties, including a high surface-area-to-volume ratio, interconnected nano-porosity, and high mass transport capabilities, thus making them highly attractive materials [2]. Incorporating biological products (bacteria, peptides, proteins, living cells, nucleic acids, viral particles, and vaccines) into fibrous scaffolds further expands the range of suitable applications and improves scaffold efficacy. For example, antibiotics are typically the first line of treatment for bacterial infections; however, with the increasing problem of antibiotic resistance in clinical therapy, research has focused on the use of probiotics to treat and prevent infection. Studies have shown the use of probiotic strains of bacteria offers many beneficial effects by inhibiting pathogen colonisation and invasion and by modulating the host immune response. However, some of the significant downfalls of commercially available probiotics are that they often contain non-viable or “dead” microorganisms that do not reach the targeted site, they show probiotic instability once at the target organ, and there is a lack of patient compliance in taking several probiotic tablets. To mitigate this, researchers have incorporated probiotic formulations into biomaterial scaffolds as they provide protection (i.e., improve probiotic viability and stability), allow their controlled release, reduce their immunogenicity, and allow for targeted release/treatment [3,4,5,6,7,8]. González et al. reported incorporating probiotics into a collagen matrix improves probiotic efficiency as it (i) promotes the formation of a probiotic biofilm, (ii) protects the live probiotic from harsh environmental conditions (both during storage and in vivo), thus improving its stability, (iii) enhances the metabolic activity of the entrapped probiotic, and (iv) enhances probiotic adhesion to the mucosa [6]. Through computational modelling, Rai et al. have demonstrated the use of sustained-release 3D-printed probiotic scaffolds improves probiotic recovery and offers patients customised therapy by altering the length of delivery [9]. Jiao et al. reported that incorporating peptides and growth factors into electrospun fibrous scaffolds improves wound recovery and tissue regeneration [10].

Both fibre production and biopharmaceuticals represent huge areas of academic and industrial interest, with the nanofibre market expected to grow by 25.1% from 2020 to 2027 [11] and the biopharmaceutical market by 7.32% from 2021 to 2026 [12]. Though scaffold fabrication is a rapidly evolving field, the techniques capable of producing functionalised fibrous scaffolds are still limited. Fibres formed from polymer solutions offer a simple way to incorporate biological products while simultaneously drying, immobilising, and solidifying the products in a gentle/non-damaging way. At present, fabrication methods include template synthesis, phase separation, self-assembly, biocomponent fibre production, melt-blowing, centrifugal spinning, and electrospinning [13]. Though these methods have advantages, most of them cannot be exploited at an industrial level since their productivity levels are low [13].

However, electrospinning has some disadvantages. For example, the variety of polymers that can be used is limited [14]. Additionally, the high voltages used can also damage biological products by disrupting cell membranes and altering the conformation of biomolecules [15,16]. Moreover, the addition of biological products can also affect the properties of the polymer solution itself, which, in turn, affects its spinnability [17]. The electrical conductivity of the solution can impact the jet formation and fibre diameter such that solutions with very high or very low conductivity cannot be electrospun [17]. The addition of bacteria and other biological products can increase electrical conductivity by introducing extracellular proteins and ions into the polymer system, as well as altering the viscoelasticity of the solution [18]. One of the drawbacks of electrospinning is its low-cost-yield efficiency. Typical single-needle setups generally produce low yields, and while multi-nozzle setups capable of higher yields exist, nozzle clogging and neighbouring jet interference are some of the disadvantages associated with it [19]. For these reasons, pharmaceutical and other industries require a production method capable of high yields to achieve sufficient production rates.

**Figure 1:**
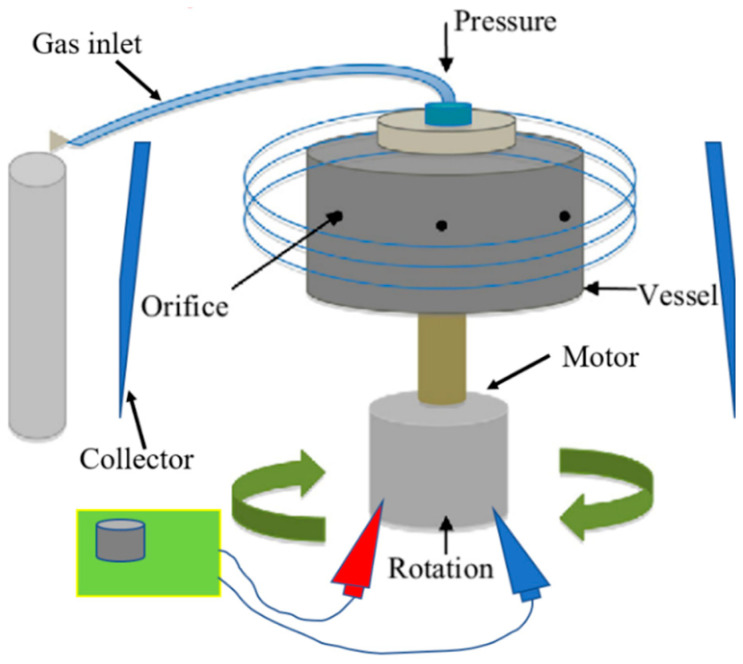
Diagrammatic representation of pressurised gyration.

Pressurised gyration (PG) is a novel, electric-field-free technique capable of producing nano- and microfibres on a large scale in a single step [20]. The setup for this process involves a perforated vessel (polymer solution is placed inside) connected to an electric motor to rotate the vessel at up to 8500 rpm, as well as a gas inlet where pressurised nitrogen gas is supplied to the vessel (Figure 1) [20]. The high-speed rotation, combined with the gas pressure, creates a large pressure differential within the vessel, which forces the polymer solution out of the vessel orifices to form a polymer jet [13,21]. A surface tension gradient occurs along the air–liquid interface, which separates the drop from the surrounding air and prompts a Marangoni stress tangential to the air–liquid interface, which causes the flow to the tip of the polymer droplet [20,21]. This jet stretches to form fibres, and as the solvent evaporates, dry fibres are deposited onto nearby walls from which they can be collected [21]. Both surface tension and solution viscosity play an instrumental role in fibre formation, particularly in pressurised gyration. During this process, the polymer solution needs to have sufficient intermolecular entanglement and chain overlap; therefore, satisfactory viscosity and surface tension at the interfacial boundary are needed to overcome the centrifugal force and dynamic fluid flow and to stabilise the polymer jet ejecting from the perforations during manufacture [22]. In general, as polymer solution surface tension and viscosity increase, so does fibre diameter [13]. However, when the surface tension and viscosity sit outside the optimal window, droplets will be formed as opposed to fibres [22,23]. This technique offers many of the same advantages as electrospinning (simple, easily modifiable, etc.), with the additional convenience of not utilising an electric field, permitting the spinning of an almost limitless selection of polymer solutions regardless of their electrical conductivity. This makes PG potentially more applicable to the incorporation of biological products. Pressurised gyration has been successfully used to manufacture a number of composite fibres for a variety of applications, including, but not exhaustive of, antimicrobial fibres for filtration [24,25,26,27], drug-loaded fibres for wound healing applications [28,29,30,31], biocompatible fibres for tissue engineering [32], and core-sheath fibres [33,34,35]. This method also shows the potential to be upscaled for industrial and commercial settings. It can produce large yields (ranging from 0.02 [36] to 6 kg/h [20]) of fibres, whereas a laboratory-scale electrospinning apparatus with a single needle can produce 0.01–2 kg/h, and commercially available industrial-scale electrospinning can produce 12 kg/h; however, there are a number of limitations associated with it, particularly when processing biologically sensitive materials.

As novel methods such as PG emerge with different potential industrial applications, there comes a need for an investigation into the best procedures for incorporating biological components and the benefits and challenges involved. The aim of this study is to provide a proof of concept for the incorporation of bacteria into microfibres using PG and to analyse the effects of the process on biological products.

## 2. Materials and Methods

### 2.1. Materials

Polyvinylpyrridone (Mw 1,300,000) (PVP), polyethylene oxide (Mw 200,000) (PEO), absolute ethanol (99%) (eth), LB agar, LB broth, and phosphate buffer saline (PBS) tablets were purchased from Sigma-Aldrich Co. Llc (Gillingham, Dorset, UK). *Bacillus subtilis* ATCC 6633 was purchased from ThermoFisher Scientific (Oxford, Oxfordshire, UK).

### 2.2. Polymer System Selection and Optimisation

Multiple polymer–solvent systems were trialled by PG to determine the most suitable systems for the scaffold (highlighted in green, Table 1). All solutions were dissolved using either a hand mixer or a magnetic stirrer for 24 h. Once completely dissolved, the solutions were processed using pressurised gyration with an applied gas pressure of 0.1 MPa, a rotation of 8500 rpm, and a consistent collection distance of 150 mm. The environmental humidity and temperature were kept at around 20–25% and 40 °C, respectively, as this was found to optimise solvent evaporation.

### 2.3. Rheological Testing

The surface tension and viscosity of the three selected polymer systems were measured using a calibrated Kruss Tensiometer K9 (Kruss GmbH, Hamburg, Germany) and a DV-III Ultra programmable rheometer (Brookfield Engineering Laboratories Inc., Middleboro, MA, USA), respectively. The surface tension of the polymer solution was measured using the Du Noüy (Ring) Tensiometry Method; the tensiometer was calibrated with water (against a reference value of 73 mN/m). Solution viscosity was calculated using concentric cylinders (also known as Couette geometry); the tensiometer was calibrated using water and ethanol before measuring each of the polymer solutions. Rheological experiments were conducted in triplicate.

### 2.4. Fibre Characterisation

Fibres formed from the selected polymer systems were examined under a scanning electron microscope (SEM) (Hitachi S-3400N, Tokyo, Japan). Samples were mounted on carbon stubs and gold sputter-coated for 90 s (using a Quorum Q150R Rotary Pumped Sputter Coater, Laughton, UK) before SEM imaging. The coated samples were imaged at an operating voltage of 5 kV. The SEM images were then analysed using ImageJ software (Version 1.53t, National Institute of Health), where 100 fibres from three separate samples were measured at random, and the average/median fibre diameter was calculated. High magnification SEM allowed for the visualisation of the fibre surface morphology.

### 2.5. Preparation of Bacteria-Loaded Fibres

*Bacillus subtilus* ATCC 6633 was used to model bacterial cells throughout these experiments. *B. subtilis* was selected as a model organism because it is an aerobic, Gram-positive bacteria found in the human gastrointestinal tract that is often used as a probiotic. As a biosafety level 1 microorganism, it is easy to work with. A working *B. subtilis* culture was prepared from a Culti-Loop, as directed by the manufacturers (LGC Standards, Teddington, UK). The culture was then streaked onto LB agar plates and incubated for 24 h at 37 °C. The plates were stored at 5 °C. A single colony was transferred into three tubes of 30 mL LB broth (one colony for each tube) and incubated overnight at 37 °C and 150 rpm. The resulting bacterial suspensions were washed once with 30 mL of PBS. The supernatant was removed, and the cells were resuspended in either (i) PBS to achieve a McFarland Standard 1 or (ii) ethanol to achieve a McFarland Standard 0.5.

To ensure proper dissolution, the polymers were pre-dissolved in the required solvent before the addition of the bacterial suspension. In total, 2.5 g of PVP was added to 8 mL of sterile PBS, 2.0 g of PEO was added to 8 mL of sterile PBS, and 2.0 g of PVP was added to 8 mL of absolute ethanol. A total of 2 mL of each bacterial suspension was then added to the polymer system (as shown in Table 2) to produce a total volume of 10 mL. The polymer systems were thoroughly mixed to ensure an even distribution of cells throughout the solutions.

Fibres were generated using PG from each of the bacterial cell-loaded polymer systems. The humidity and temperature were kept at ~20–25% and 40 °C. The product from 10 mL of each solution was collected and weighed for yield calculations. Aseptic techniques were used to ensure the spinning area was kept sterile.

### 2.6. Viability Testing and Imaging

The fibres generated from each polymer system were deposited into 30 mL of LB broth and incubated at 37 °C and 150 rpm overnight. The resulting suspensions were visually analysed for turbidity to indicate growth. The number of *B. subtilis* cells in each suspension after incubation was calculated using serial dilution and colony count. The number of colonies formed was counted to determine the viability of the cells released from the scaffolds. Bacterial cell viability studies were conducted in triplicate. PEO/PBS bacterial-loaded fibres were imaged using SEM (GeminiSEM 360, Carl Zeiss Microscopy GmbH, Oberkochen, Germany) with a 1 kV acceleration voltage.

## 3. Results and Discussion

### 3.1. Polymer System Selection and Analysis

A variety of polymers and solvents were trialled to determine their suitability for the desired application (Table 3). When working with highly sensitive live cells, water-based solvents are preferred as they are conducive to cell viability and would allow for an accurate test of PG’s effect on cells (instead of assessing the solvent’s effect on cell viability). For this reason, focus was placed on water-soluble (hydrophilic) polymers. PBS was ultimately used as a solvent as it is isotonic and, therefore, would not cause swelling or bursting of the cells when in contact with the polymer solution phase (before spinning) while providing the desired qualities of water. Ethanol was also investigated as a possible solvent due to its low toxicity compared to other solvents [37]. It also has weaker intermolecular bonds compared to water, which allows for faster evaporation. These selections are highly compatible with the PG process and generate a high yield of fibres. Although ethanol is known to disrupt the physical structure of cell membranes [28], it was still included within a selected polymer system due to its positive effect on fibre yield [36].

PVP and PEO were selected as polymers due to their solubility, biocompatibility, biodegradability, non-toxicity, and their FDA approval as drug delivery agents [38]. PVP has a successful history of usage in tissue engineering in areas such as bone tissue engineering and scaffold fabrication [39]. It is also commonly used within the pharmaceutical industry as a stabiliser, crystallisation inhibitor, and solubility enhancer for poorly soluble drugs and is readily soluble in solvents with a hydroxyl group [38,40]. PEO is also a widely used polymer in drug delivery and tissue engineering [41]. It has been used as a copolymer and carrier polymer in drug delivery systems and has been combined with other polymers for use in hard and soft tissue engineering [42,43,44,45]. PVP and PEO with Mws of 1,300,000 and 200,000 Da, respectively, were used to maximise processibility and fibre yield due to their high degrees of chain entanglement [46]. These polymers with the same molecular weights have been utilised in PG in other works and have resulted in fibres being produced [42,47]. Polymer system selection affects the final application of the scaffold as the biocompatibility of the scaffold itself and its dissolution products depend on the polymer–solvent reactions present within the system. The release rate of the biological products is dependent on their distribution within the nanofibres and the nature of the polymer [18]. Cell encapsulation within nanofibres requires a comprehensive understanding of how the polymer will interact with its environment and also how the solvent will interact with the biological products being encapsulated.

The rheological properties of each polymer solution are shown in Table 3. The surface tension of the polymer system impacts the minimum rotational speed and applied gas pressure the PG system must operate at to produce fibres. Since the centrifugal force produced by the system must be large enough to overcome the surface tension of the polymer solution, higher rotational speeds and gas pressures are therefore required for fibre production to occur [13]. The surface tension of a polymer solution is largely determined by the solvent and will closely resemble the surface tension of the selected solvent [48]. For the PVP/PBS and PEO/PBS systems, their average surface tensions were 73.2 and 73.9 mN/m, respectively. These measurements closely resemble the surface tension of water (72.8 mN/m) [49]. The surface tension of the PVP/ethanol polymer system was measured as 22.6 mN/m, very close to the surface tension of ethanol (22.1 mN/m) [50]. This indicates that using water as a solvent within polymer systems for PG will produce lower fibre yields compared to using ethanol, which evaporates more easily than water.

Polymer concentration is the main factor that influences fibre formation and morphology. Viscosity and chain entanglement are correlated to polymer concentration. The PVP/PBS 25% system had the highest viscosity and produced very fine, highly entangled fibres, indicating it has sufficient surface tension and viscosity at its interfacial boundary to overcome the centrifugal flow. The fibres produced from this system were also sticky and wet. This was due to the system’s high viscoelasticity, which creates a dense polymer chain that can prevent the travel of solvent within the highly congested polymer matrix [51]. This results in irregular solvent evaporation and, thus, wet and sticky fibres with visible water droplets. The PEO/PBS 20% and PVP/ethanol systems had lower viscoelasticity and, therefore, stronger and dryer fibres were produced using these systems. The PEO/PBS system contains a lower concentration of polymer of a lower molecular weight, meaning there is less chain entanglement and less solvent is prevented from evaporating. The PVP/ethanol system contains a low polymer concentration and an easily evaporating solvent when compared to PVP/PBS 25%, which minimised the amount of irregular solvent evaporation and created stronger and dryer fibres. The structural integrity of the produced fibres has a large impact on the protection afforded to the biological products by the polymeric scaffold; therefore, the effect of the viscoelasticity of the polymer solution needs to be carefully selected.

### 3.2. Fibre Characterisation

**Figure 2:**
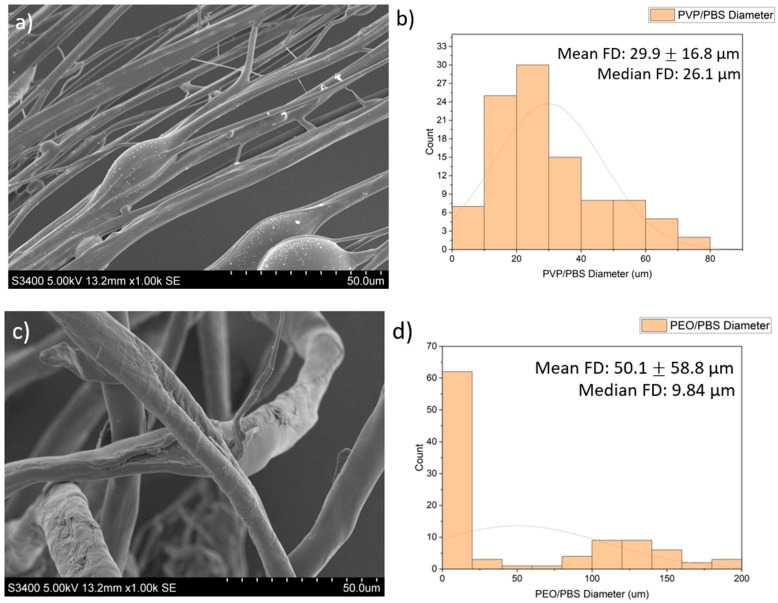

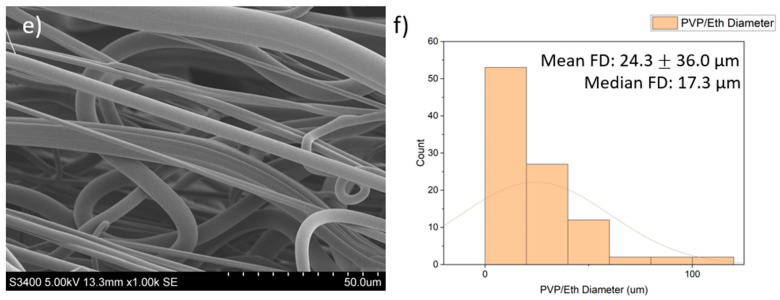
Images of the PG fibres: (**a**) SEM images of PG PVP/PBS 25% fibres (scale bar = 50.0 μm); (**b**) histogram showing distribution of PG PVP/PBS 25% fibres (*n* = 100) with a line of best fit; (**c**) SEM images of PG PEO/PBS 20% fibres (scale bar = 50.0 μm); (**d**) histogram showing distribution of PG PEO/PBS 20% fibres (*n* = 100) with a line of best fit; (**e**) SEM images of PG PVP/ethanol 20% fibres (scale bar = 50.0 μm); (**f**) histogram showing distribution of PG PVP/ethanol 20% fibres (*n* = 100) with a line of best fit.

SEM images of the fibres are presented in Figure 2. The PVP/PBS 25% fibres had a mean fibre diameter (FD) of 29.9 μm (±16.8 µm) and median FD of 26.1 μm, with a large amount of variation in the size and morphology of fibres. The fibres appeared smooth and cylindrical with some beads present. The PEO/PBS 20% fibres measured a mean FD of 50.1 μm (±58.8) and median FD of 9.84 μm, with a large amount of variation in the size and morphology of the fibres. The fibres have a rough topography with a lot of overlapping, but no beads were present. The PVP/ethanol 20% fibres measured a mean FD of 24.3 μm (±36.0) and median FD of 17.3 μm with a large amount of variation in the fibres. The fibres appear to be normally distributed with some fibres having very large diameters. The fibres appear smooth and cylindrical with some beads present.

The diameters and diameter distribution heavily influence the dissolution pattern of the scaffold, which impacts biological release and the structural integrity of the scaffold. The PVP/PBS 25% and PVP/ethanol 20% polymer systems produced fibres with average diameters of 29.9 μm and 24.3 μm, respectively, and an approximately normal distribution with relatively few fibres with very large diameters. Although the fibres produced by PEO/PBS 20% had a larger mean fibre diameter (50.1 μm), the median fibre diameter was 9.84 μm, indicating this polymer system produced the smallest fibres as there were clear outliers in the fibre pool that skewed the mean of the fibre diameters. According to Figure 2c, most fibres produced from PEO/PBS have extremely small diameters, while most fibres produced by the PVP/PBS and PVP/ethanol systems were of a larger size (Figure 2a,e). The PVP used within the polymer systems had a very large molecular weight (1,300,000 Da), and the PEO used had a lower molecular weight (200,000 Da), contributing to the different fibre morphologies seen. Between the two polymer systems produced using PVP, the PVP/ethanol system appeared to produce finer fibres. This is because as solvent volatility increases, the fibre diameters decrease, owing to more rapid evaporation [11]. Ethanol has weaker intermolecular bonds than water, making it more volatile and allowing it to evaporate faster, resulting in the observed fibre morphologies.

The yield for the PVP/ethanol polymer system was greater than the other systems utilising PBS (Table 4). This is because ethanol is a much more volatile solvent and evaporates faster than PBS, allowing for more fibres to be formed and deposited on the collector.

### 3.3. Bacterial Cell Viability

**Figure 3:**
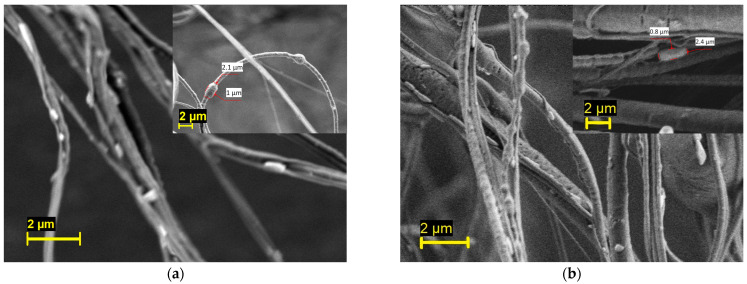
Scanning electron micrograph of the (**a**) PEO/PBS and (**b**) PVP/PBS bacterial-loaded fibres.

The PEO/PBS and PVP/PBS bacterial-loaded fibres were imaged using SEM, as shown in Figure 3. Generally speaking, fully mature *B. subtilis* cells are rod-shaped cells that are 2 to 6 µm in length and less than 1 µm in diameter [52]. In this figure, rod-shaped cells can be seen on the fibre surface, thus confirming the successful incorporation of bacteria into the fibres. The inset of the micrograph also depicts some bacteria trapped in the fibres. This result indicates that PG is a promising technique for fabricating fibres containing viable microorganisms.

**Figure 4:**
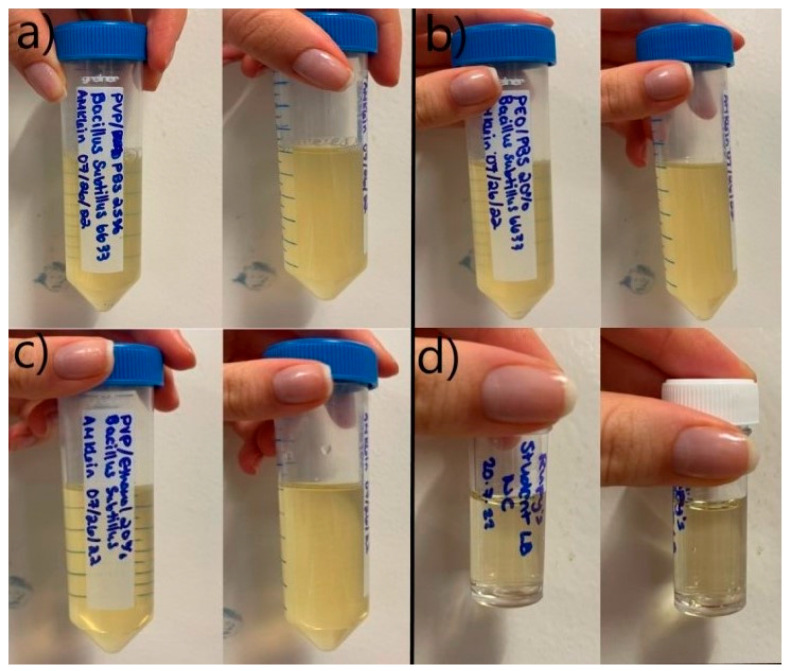
Overnight bacterial growth from fibres; (**a**) PVP/PBS 25% fibres; (**b**) PEO/PBS 20% fibres; (**c**) PVP/ethanol 20% fibres; (**d**) negative control (no fibres present).

The bacterial cell-loaded polymeric fibres were collected and incubated overnight in LB broth to assess the release and viability of the encapsulated cells. Following overnight incubation, microbial growth was assessed qualitatively and quantitatively. A qualitative assessment of the media showed the broth containing PVP/PBS and PEO/PBS fibres produced high microbial turbidity (Figure 4a). High turbidity indicates a high cell density within the culture sample. The PVP/ethanol fibres showed no turbidity and, therefore, no/little bacterial growth can be expected. A negative control with no fibres was used to test for broth contamination, which produced no turbidity and, therefore, no bacterial growth.

Microbial growth occurred from the PVP/PBS fibres and the PEO/PBS fibres but not from the PVP/ethanol fibres. The PVP/PBS fibres produced more microbial growth than the PEO/PBS fibres. This could have been due to the different polymer concentrations and molecular weights of the systems. Higher polymer concentrations with longer chain lengths allow for more chain entanglement to occur and encapsulate the materials within the polymer solution. This results in a higher encapsulation efficiency and, thus, more microbes being released. PVP is known to be an effective stabilising agent by reinforcing intermolecular bonds between various drugs and carriers, which has been correlated with PVP’s antiplasticising effect, ability to form hydrogen bonds, and hydrophilicity enhancement [40]. These characteristics likely enabled this polymer to form fibres that protect and encase the incorporated bacteria effectively and release a larger amount of viable cells into the environment. This is likely due to the cells coming into direct contact with ethanol, which disrupts cell membranes and is known to be effective at killing microorganisms [53]. This direct contact likely resulted in the increased permeability of the cells’ membranes, allowing substances to leak into and out of the cell [53]. This is because ethanol is capable of dissolving non-polar substances like the lipids that make up cell membranes [54]. Ethanol was selected as a solvent for processing because it was unknown whether the short contact time during PG would affect the cells and to what degree. This finding indicates that solvent selection for this application will greatly influence the viability of the biological products/biopharmaceuticals, even when working with very short processing times. This could limit the number of polymer–solvent solutions that can be used for biopharmaceutical delivery, as many solvents typically used in PG (DMF, methanol, ethanol, etc.) might not be compatible and cause damage to cells. It is unlikely that the polymer choice affected the viability of the cells because growth resulted from the PVP/PBS fibres. This indicates the solvent choice had a greater effect and was likely a determining factor for cell viability than the polymer choice.

*B. subtilis* forms colonies that can be described as rough, opaque, and fuzzy white or slightly yellow with jagged edges [55], which match the description of the colonies formed from the PVP/PBS and PEO/PBS cell-encapsulating fibres. The colonies produced indicate that the encapsulated cells were able to survive the forces they underwent during PG. These cells were subjected to rotational speeds of up to 8500 rpm and 0.1 MPa of gas pressure to encapsulate them into fibres, although they were subjected to this force for a very short period (less than 1 min), which could have allowed them to survive the extreme forces. The colonies formed from the two sets of PBS-spun fibres also produced a similar number of colonies, which was expected because the polymer solutions contained approximately the same number of CFUs (both were produced from McFarland standard 1 bacterial suspension within PBS and were inoculated with about ${6.0\times 10}^{8}$ CFU each). These results indicate that cells and other biological products can survive the PG process and be incorporated into fibres using PG.

This study is the first to evaluate the viability of the PG process for the incorporation of cells and other biological products. Currently, industrial-scale electrospinning has some limitations, including lengthy processing times, electrostatic field interaction between needles, needle clogging, and limited material options (i.e., electrospinning nanofibres from natural materials with poor mechanical properties, such as silk fibroin, may need special treatment with organic and caustic solvents to increase their stability, which creates safety and environmental considerations) [56].

Production methods that use centrifugal force instead of electrostatic forces have shown a potential to become a viable alternative to electrospinning for the mass production of fibres that can safely, consistently, and cost-effectively be upscaled. PG can produce fibres in a simple and rapid process independent of a magnetic or electric field, resulting in lower production costs and the ability to process sensitive materials [32]. Throughout this process, the polymer solution and incorporated material are subjected to intense centrifugal force and pressure to overcome the solutions’ surface tension and produce fibres; however, the effect this has on living cells is largely unknown. Most biological product-incorporated fibres are intended as alternative delivery vehicles for medical therapies, where they provide protection and a template for growth, as well as utilise the fibres’ large surface-to-volume ratio to improve delivery [18]. The uses for cell-loaded fibres extend to areas such as probiotic delivery, agriculture (fungus and cyanobacteria), and stem cell delivery (tissue engineering and wound healing) [18]. These areas represent therapeutic and economic potential but are drastically limited by the ability of nanofibre-production methods to be scaled up to an industrial/commercial scale. If fibres are to become globally accessible to the public, multiple cost-effective and efficient techniques will need to be available to lower production costs, and the compatibility for cell-incorporation using these methods should be thoroughly explored and understood. From this study, it can be determined that bacterial cells are able to survive and remain viable throughout the PG process.

## 4. Conclusions

Multiple polymer systems were trialled to generate fibres by PG, and the rheological properties of the selected polymer systems were measured. It was found that the surface tension and viscosity heavily influenced the spinnability of the solution and the morphology of the fibres produced due to the different levels of chain entanglement and solvent evaporation. Fibres produced using PEO appeared to produce the thinnest fibres, while PVP produced thicker fibres, largely due to the difference in the polymer molecular weights. Cell-loaded fibres were produced using the selected polymer systems and placed in LB broth overnight to determine the viability of the cells released from the fibres. It was found that only the PVP/PBS 25% and PEO/PBS 20% fibres released viable cells, whereas the PVP/ethanol 20% fibres did not, likely due to the solvent’s effect on the cells and their membranes. From analysing colonies produced from the cells released from the fibres, it was determined that the cell-loaded fibres could release biologically active cells and that PG represents a promising method for incorporating biological products into fibres. The scaffolds developed in this study offer a proof of concept that PG can be used to produce cell-loaded fibres and opens the door for the incorporation of many other microorganisms/biological products for a wide variety of applications. This study uncovers an additional dimension of the utility of PG, which could help unlock the potential of fibres in probiotic delivery, agriculture, and stem cell delivery and make them accessible on an industrial scale.

## Figures and Tables

**Table 1:** List of polymer systems trialled for fibre production.

Polymer/Concentration (*w*/*v*)	Molecular Weight (Mw)	Solvent	Fibres Produced
PVP 20%	1,300,000	Ethanol	Yes—very strong yield of fibres
PVP 20%	1,300,000	PBS	No—yield insufficient
PVP 30%	1,300,000	Ethanol	No—far too viscous
PVP 25%	1,300,000	PBS	Yes—good yield + wet fibres
PVP 30%	1,300,000	PBS	Yes—very small yield
PVA 20%	31,000–50,000	Ethanol	No—did not dissolve at all
PVA 20%	31,000–50,000	PBS	No—did not dissolve sufficiently
PVA 10%	31,000–50,000	PBS	No—dissolved but no fibres
PVA 10%	146,000–186,000	PBS	No—dissolved but no fibres
Gelatine 7.5%	Gel strength 300, Type A	PBS	No—stayed jelly-like in pot
PEO 20%	200,000	PBS	Yes—lots of fibres—quite delicate + stretchy
PEO 20%	1,000,000	PBS	No—did not dissolve (too viscous)

**Table 2:** Table showing amount of colony forming units (CFUs) that were incorporated into each polymer system.

Suspension	Avg. Absorbance 600 nm	McFarland Standard of Solvent Suspension	Approximate Cell Density (1 × 10^8^ CFU/mL)	Volume Added to Polymer System (mL)	CFU Added to Polymer System
1 (PVP/PBS 25%)	0.2830	1	3.0	2.0	${6.0\times 10}^{8}$
2 (PEO/PBS 20%)	0.2785	1	3.0	2.0	${6.0\times 10}^{8}$
3 (PVP/Eth 20%)	0.0994	0.5	1.5	2.0	${3.0\times 10}^{8}$

**Table 3:** Rheological properties of the selected polymer systems (*n* = 3).

Polymer System (*w*/*v*)	Surface Tension (mN/m)	Viscosity (mPa·s)
PVP/PBS 25%	73.4 ± 0.5	3659.0 ± 82.4
PEO/PBS 20%	73.9 ± 0.4	1513.0 ± 60.5
PVP/Ethanol 20%	22.6 ± 0.1	1132.0 ± 55.4

**Table 4:** Product yield and microbial growth resulting from the produced bacteria-loaded fibres (*n* = 3).

Polymer System	Yield of Collected Fibres (%)	Starting Microbial Concentration in Scaffold (CFU/mL)	Microbial Concentration after 24 h (CFU/mL)	$\mathrm{Microbial}\;\mathrm{Growth}\;(\%)\;(\frac{Starting\;conc.}{Conc.after\;24\;h})\times 100\%$
PVP/PBS 25%	1.2	${3.0\times 10}^{8}$	${1.8\times 10}^{8}$	60 ± 2
PEO/PBS 20%	3.1	${3.0\times 10}^{8}$	${1.4\times 10}^{8}$	47 ± 4
PVP/Ethanol 20%	4.5	${1.5\times 10}^{8}$	0	0 ± 0

## Data Availability

Data is contained within the article.
